# Treatment outcomes in lower limb lymphedema misdiagnosed as iliac vein compression syndrome: A retrospective analysis

**DOI:** 10.1371/journal.pone.0323077

**Published:** 2025-10-17

**Authors:** Guijun Huo, Mingqing Du, Zhichao Yao, Dayong Zhou, Zhanao Liu

**Affiliations:** Department of Vascular Surgery, The Affiliated Suzhou Hospital of Nanjing Medical University, Suzhou Municipal Hospital, Gusu School, Nanjing Medical University, Suzhou, China; Ataturk University Faculty of Medicine, TÜRKIYE

## Abstract

Iliac vein compression is highly prevalent in the general population, which may lead to misdiagnosis of lower limb lymphedema as iliac vein compression syndrome and subsequent stent placement. This study retrospectively analyzed the treatment outcomes of 11 patients with secondary lymphedema who had previously been diagnosed with iliac vein compression by venography and underwent iliac vein stenting. Following iliac vein stent placement, six patients with Stage I and IIa lymphedema experienced partial relief of limb swelling; however, symptoms recurred and worsened within three months. The remaining patients showed no improvement in swelling after the stent was placed. Due to inadequate symptom relief following stent implantation, these patients underwent reevaluation and were subsequently diagnosed with lymphedema. Based on disease staging, they received appropriate interventions including complex decongestive therapy, lymphovenous anastomosis, or a combination of liposuction and lymphovenous anastomosis. Four patients with Stage I and IIa lymphedema underwent complex decongestive therapy, four patients with Stage I and IIa lymphedema received lymphovenous anastomosis, and the remaining three patients with Stage IIb lymphedema underwent liposuction combined with secondary lymphovenous anastomosis. Follow-up assessments were conducted at 3, 6, and 12 months post-treatment to evaluate limb morphology and functional outcomes using the Disability and Health Questionnaire for Lower Limb Lymphedema scores. Therapeutic outcomes analysis revealed that complex decongestive therapy, lymphovenous anastomosis, and liposuction demonstrated favorable efficacy in managing lymphedema cases with suboptimal response to prior iliac vein stenting.

## Introduction

The escalating incidence of malignant tumors and the enhanced survival rates of patients following radical tumor surgeries in recent years have led to a notable surge in lymphedema occurrence [1]. Patients presenting with lower limb lymphedema at outpatient clinics may be misdiagnosed with edema resulting from other causes, such as cardiogenic, renal, or venous edema [2,3]. In clinical practice, lower limb lymphedema is frequently misdiagnosed as edema of other etiologies due to overlapping symptoms and the lack of lymphatic imaging techniques during initial evaluation.

Iliac vein compression syndrome is a common disease in vascular surgery. Previous studies have reported that the incidence of iliac vein compression is exceptionally high in the general population, reaching as high as 65% [4,5]. The left common iliac vein often traverses the space between the aorta and lumbar vertebrae, and its compression can result in symptoms of venous outflow obstruction [6,7]. This syndrome is particularly prevalent among middle-aged women between 30 and 50 years of age [8]. Patients in this age group who develop lower limb edema following surgery for cervical or endometrial cancer are often referred to vascular surgery or interventional radiology departments. These patients typically undergo lower limb venography, and those found to have iliac vein compression may be diagnosed with iliac vein compression syndrome and subsequently treated with stent placement. The overlap in clinical presentation between lymphedema and venous edema can lead to diagnostic challenges, delaying appropriate lymphatic management.

In clinical practice, patients with lower limb lymphedema are occasionally diagnosed with iliac vein compression through imaging examinations and subsequently undergo stent placement. By summarizing various treatment approaches and their corresponding outcomes, this study aims to highlight the importance of accurate differential diagnosis and the necessity of adopting an integrated management strategy in such cases.

## Methods

### Patients

This study was conducted by the principles outlined in the Declaration of Helsinki. Approval was granted by the Ethics Committee of Suzhou Municipal Hospital (IRB Number: J-2024–042-K01, dated November 25, 2024), and individual consent, including for photograph acquisition and distribution, was obtained from all included patients. The data utilized in this study were collected on February 25, 2025. The authors had access to participants’ identifiable information during the data collection phase. From January 2022 to June 2024, 11 patients who had initially presented with lower limb swelling, were diagnosed with iliac vein compression syndrome, and underwent iliac vein stenting, were subsequently admitted to our department due to disease progression and diagnosed with lymphedema. The diagnosis of iliac vein compression was established through lower limb venography, demonstrating ≥50% luminal stenosis with extensive collateral vein formation. Among these cases, 10 patients who received iliac vein stenting at external hospitals underwent ^99^Tc^m^-DX lymphoscintigraphy only after being transferred to our institution, with no pre- or post-stenting lymphatic imaging performed at their original hospitals. The patient inclusion criteria were confirmation of lymphatic obstruction through ^99^Tc^m^-DX lymphoscintigraphy, presence of unilateral secondary lymphedema, and a minimum 10% increase in the affected limb volume compared to that of the contralateral side. The patient exclusion criteria consisted of deep venous thrombosis or iliac vein stent occlusion, inability to comply with compression therapy measures, age > 80 years, recurrence of malignant tumors, and those who were lost to follow-up. The demographic information, limb circumferences, cancer surgical approach, chemical and radiation therapy, and erysipelas episodes of all included patients were recorded.

### Pre-treatment assessment

A medical history inquiry and physical examination were conducted to determine the initial diagnosis and staging of the patient’s lymphedema, strictly adhering to the staging system proposed by the International Society of Lymphology (ISL) (Table 1) [9,10]. The morphological indicators of the patient’s limbs before iliac vein stent implantation and the effect and duration of postoperative detumescence were also investigated. Complete resolution of the affected limb to symmetry with the contralateral limb after iliac vein stent placement was defined as disease remission, whereas other situations were regarded as partial remission or ineffective treatment. All patients underwent ^99^Tc^m^-DX lymphoscintigraphy to confirm lymphatic obstruction and were screened for deep venous thrombosis or stent occlusion via vascular ultrasound or phlebography of the lower extremities. Furthermore, a limb magnetic resonance imaging scan was performed to determine whether the affected limb had fluid-predominant or solid-predominant lymphedema [11]. Limb morphology measurements were also conducted before the patient underwent treatment to assess the severity of the condition, with all limb circumferences calculated at the midpoint, lower third, and upper third of the calves and thighs. Additionally, the frustum method was applied to estimate the lower limb volumes [12]. Lastly, the Lymphedema Functioning, Disability and Health Questionnaire for Lower Limb Lymphedema (Lymph-ICF-LL) score and limb circumference/volume measurements were employed to establish the baseline status of the disease [13]

**Table 1:** Staging system of the international society of lymphology [9,10].

International Society of Lymphology Staging System	
0	Latent or subclinical lymphedema
I	Lymphedema that subsides with limb elevation
IIa	Lymphedema does not subside with limb elevation, and pitting edema is observed
IIb	Pitting edema is difficult to detect, while fibrosis and adiposity become prominent
III	Lymphostatic elephantiasis. Pitting edema is absent. Advanced stages of adiposity, fibrosis, and dermal thickening with warty overgrowths

### Surgical treatment

**Fig 1:**
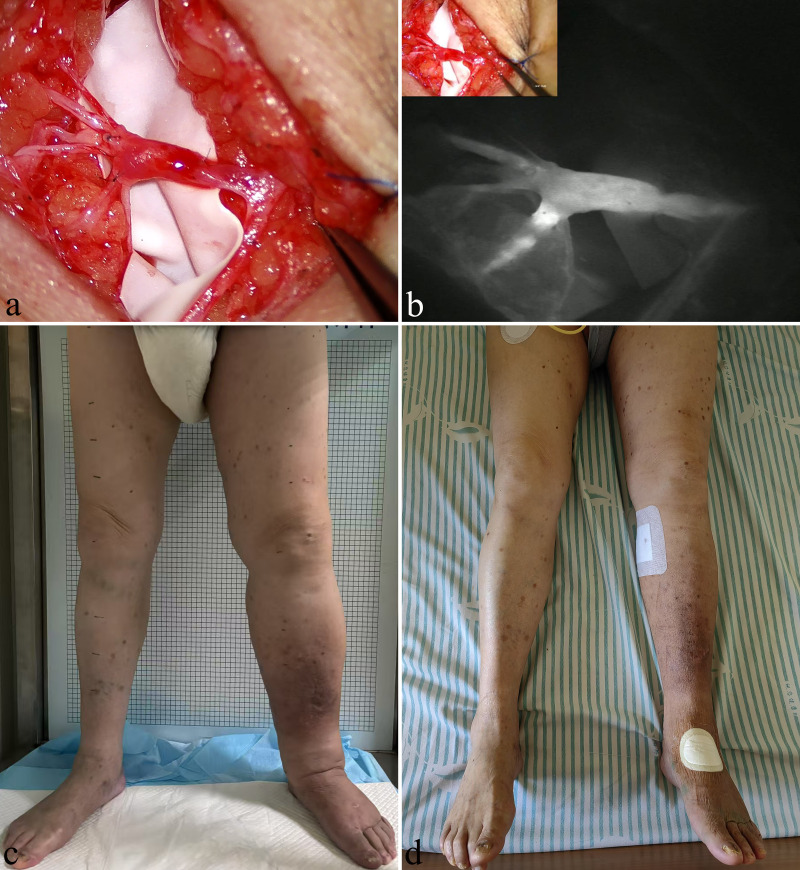
A 60-year-old man with a history of renal tumor surgery and persistent left lower limb edema for 3 years after a prior diagnosis of left iliac vein compression syndrome and iliac vein stent implantation 1 year ago. (A) An incision was made on the medial side of the left calf. The valve function of the second-order branch of the great saphenous vein was found to be good, and seven lymphatic vessels were anastomosed into the vein. (B) Fluorescence microscopy imaging after the preoperative injection of indocyanine green for lymphangiography provided clear visualization, demonstrating a smooth entry into the veins and confirming a patent anastomosis site. (C) Preoperative assessment indicated that the left lower limb was swollen, with an International Society of Lymphology Stage of IIa. (D) On the second day after surgery, the limb swelling had significantly improved without the need for complex decongestive therapy.

**Fig 2:**
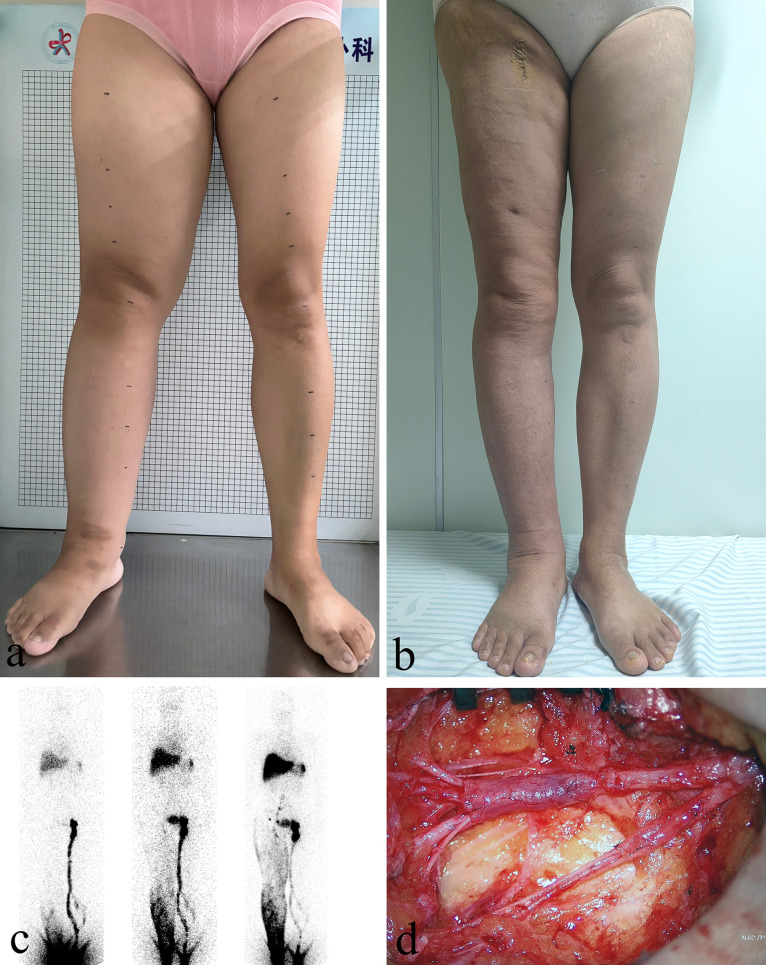
A 50-year-old woman with a history of cervical cancer surgery was diagnosed with right iliac vein compression 4 years ago and underwent stent implantation with no swelling relief. (A) Prior to the surgical procedure during the hospitalization stage, the patient’s right lower limb showed significant swelling that was classified as International Society of Lymphology Stage IIb. (B) After liposuction followed by lymphovenous anastomosis 3 months later, the swelling in the patient’s limb significantly improved. (C) Preoperative ^99^Tc^m^-DX lymphoscintigraphy in the anterior position at 10 min, one hour, and three hours post-injection demonstrated lymphatic return obstruction in the right lower limb. (D) The lymphatic vessels, the lateral femoral vein, and its branches were dissected at the inguinal level. After confirming good valve function, the lymphatic vessels were anastomosed to the lateral femoral vein and its branches.

Patients with fluid-predominant Stage I and IIa lymphedema were initially treated with complex decongestive therapy (CDT) in the lymphedema care clinic. Subsequently, patients with unsatisfactory treatment outcomes underwent peripheral lymphovenous anastomosis (LVA) after hospitalization to improve lymphatic return. LVA surgery typically involves multiple incisions, during which superficial functional lymphatic vessels are anastomosed with small veins of matching diameter under the guidance of indocyanine green lymphangiography (Fig 1A, 1B). Morphological data of the limbs before and after LVA surgery were recorded (Fig 1C, 1D). In patients with solid-predominant Stage IIb and III lymphedema, liposuction was first performed for volume reduction, followed by a second-stage LVA to enhance lymphatic return (Fig 2A, 2B). Liposuction was conducted using the tumescent technique and the power-assisted liposuction device (Yangguangzhongtian, Shanxi, China) with 35 cm long cannulas. During the 3 months following the liposuction procedure, all patients wore 30–40 mmHg compression garments for limb compression. At 3 months after liposuction, LVA was performed at the inguinal region (Fig 2C, 2D). Patients were provided short-stretch bandage compression for at least 3 months after LVA, followed by the application of compression garments with a pressure of 30–40 mmHg for maintenance.

### Follow up

During discharge, all complications were comprehensively recorded for each patient and categorized into major complications requiring surgical intervention and minor complications that could be alleviated through conservative treatment. All postoperative patients and those receiving compression therapy in the outpatient setting underwent follow-up assessments at 3, 6, and 12 months. During these assessments, the patients received guidance on compression therapy, underwent Lymph-ICF-LL scoring, and had their limb morphology measurements recorded. The volume difference between bilateral limbs and the difference rate after the treatment were also compared. The volume difference rate of the bilateral limbs before and after lymphedema treatment was defined as follows: (volume of the affected limb − volume of the healthy limb)/volume of the healthy limb.

### Statistical analysis

Statistical analysis was performed using SPSS statistical software (version 17; SPSS, Chicago, Illinois). Kolmogorov-Smironov test was used to confirm data normality. Quantitative variables were summarized as mean and standard deviation (SD) if normally distributed or median and interquartile range (IQR). Categorical variables were presented as numbers and percentages. Pre and post-treatment Lymph-ICF-LL was analyzed with paired t-tests. A value of *P* ≤ 0.05 was considered to be significant.

## Results

**Fig 3:**
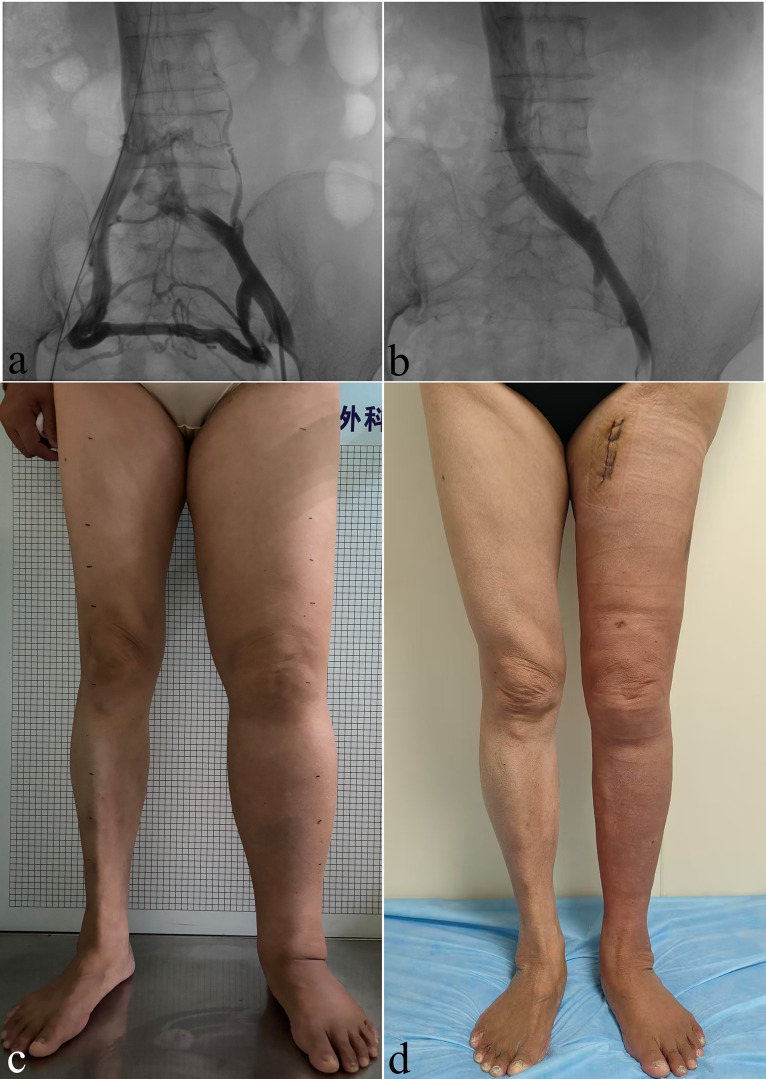
A 40s-year-old woman who had a history of cervical cancer surgery was diagnosed with left iliac vein compression syndrome 18 months earlier and underwent stent implantation. However, the swelling symptoms did not show any improvement. (A) Left iliac venography revealed severe stenosis of the left common iliac vein, accompanied by extensive collateral vessel formation, with blood flow compensation via the contralateral internal iliac vein. (B) Follow-up venography revealed smooth blood flow in the left common iliac vein after stent implantation and the disappearance of the collateral veins. (C) Before the surgical procedure during the hospitalization stage, the patient’s left lower limb demonstrated significant swelling that was categorized as International Society of Lymphology Stage IIb. (D) This female patient experienced an immediate reduction in the volume of her swollen limb after undergoing liposuction.

A total of 11 patients were included in this study, with an average age of 63.18 ± 9.89 years, and consisting of nine women and two men. The baseline characteristics of the patients are presented in Table 2. None of the patients had undergone systematic CDT prior to hospitalization. Moreover, none of the patients exhibited signs of superficial varicose veins, lipodermatosclerosis, or ulcers. Ten patients underwent iliac vein stent implantation in an external hospital, while one received the procedure in the vascular surgery department of our hospital (Fig 3A, 3B). Specifically, nine patients were treated with left iliac vein stent implantation due to left iliac vein stenosis, and two underwent stent implantation for right iliac vein stenosis. Among the patients, six Stage I and IIa lymphedema experienced partial relief of limb swelling following iliac vein stent implantation; however, all exhibited recurrence and further progression within 3 months. The remaining five patients demonstrated no improvement in limb edema after stent placement. The clinical data of all 11 patients are provided in Table 3.

**Table 2:** Demographics and baseline characteristics of the study patients.

Patient number	Gender	BMI (kg/m^2^)	Affected limb	Primary malignancy	Radiation therapy	Lymphedema duration (months)	Cellulitis history
1	Female	24	Left	Cervical cancer	Not performed	45	Single episode
2	Female	25.5	Left	Cervical cancer	Not performed	60	Never
3	Female	25.2	Right	Cervical cancer	Performed	60	Recurrent episode
4	Male	24.5	Left	Renal cell cancer	Not performed	36	Recurrent episode
5	Female	24.4	Left	Endometrial cancer	Not performed	12	Never
6	Female	18.7	Left	Endometrial cancer	Not performed	10	Single episode
7	Female	24.7	Left	Cervical cancer	Not performed	24	Never
8	Female	24.6	Right	Cervical cancer	Performed	120	Recurrent episode
9	Female	29.3	Left	Rectal cancer	Not performed	6	Never
10	Female	21.1	Left	Cervical cancer	Not performed	14	Single episode
11	Male	24.2	Left	Rectal cancer	Performed	36	Never

BMI, body mass index.

**Table 3:** Clinical data of the study patients with secondary lymphedema complicated by iliac vein compression.

Patient number	ISL Stage	Duration after stent implantation (months)	Relief of swelling after stent implantation	Relief duration (months)	Treatment approach	ICG-L	Surgical details	Reduction in affected limb volume (ml)
1	IIb	18	No	0	Liposuction+LVA	Stardust and/or Diffuse pattern	Inguinal LVA	2234
2	IIb	30	No	0	Liposuction+LVA	Stardust and/or Diffuse pattern	Inguinal LVA	2570
3	IIb	48	No	0	Liposuction+LVA	Stardust and/or Diffuse pattern	Inguinal LVA	2229
4	IIa	12	No	0	LVA	Linear pattern + Stardust pattern	2 incisions, 5 anastomoses	3204
5	IIa	7	partial relief	1	LVA	Linear pattern + Stardust pattern	3 incisions, 8 anastomoses	1095
6	IIa	5	partial relief	2	LVA	Linear pattern + Stardust pattern	2 incisions, 8 anastomoses	607
7	IIa	6	No	0	LVA	Linear pattern + Stardust pattern	3 incisions, 6 anastomoses	1241
8	IIa	24	partial relief	3	CDT	Linear pattern + Stardust pattern	N/A	1462
9	IIa	5	partial relief	3	CDT	N/A	N/A	1378
10	I	9	partial relief	2	CDT	N/A	N/A	156
11	IIa	33	partial relief	1	CDT	N/A	N/A	467

ISL, International Society of Lymphology; ICG-L, indocyanine green lymphangiography; CDT, complex decongestive therapy; LVA, lymphovenous anastomosis; N/A, not applicable.

All included patients were confirmed to have patent iliac vein stents via ultrasonography or angiography and were diagnosed with lymphedema through ^99^Tc^m^-DX lymphoscintigraphy. Four patients with Stage I and IIa lymphedema achieved satisfactory outcomes with CDT, thereby not requiring further surgical intervention. These four patients showed a median volume reduction of 923.26 (234.11, 1441.28) ml and a decrease in the median volume difference (volume difference rate) from 1469.16 mL (22%) to 545.90 mL (8%), along with a decline in the median Lymph-ICF-LL score from 155 to 56 that indicated an improved quality of life.

Four other patients with Stage I and IIa lymphedema who responded poorly to CDT underwent LVA during their hospitalization, with no major or minor complications observed postoperatively. These patients experienced a median volume reduction of 1168.72 (729.53, 2714.14) ml over a follow-up period of 6 months and a decrease in the median volume difference (volume difference rate) from 1904.30 mL (30%) to 735.58 mL (12%), while their median Lymph-ICF-LL score decreased from 168 to 66.

The remaining three patients with Stage IIb lymphedema were admitted for surgical intervention after unsatisfactory results from CDT. The surgical approach involved initial liposuctionl (Fig 3C, 3D), followed by a second-stage LVA after 3 months to improve lymphatic drainage. During the 6-month follow-up period, these patients presented with a median volume reduction of 2234.31 ml, a decrease in the median volume difference (volume difference rate) from 3013.82 mL (43%) to 784.39 mL (11%), and a decline in the median Lymph-ICF-LL score from 158 to 59. Follow-up data of all included patients are presented in Table 4.

**Table 4:** Clinical data of the bilateral limbs of the study patients before and after lymphedema treatment.

	CDT (n = 4)			LVA (n = 4)			Liposuction+LVA (n = 3)		
**Volume difference between the bilateral limbs before and after lymphedema treatment (ml)**									
Time point	Min	Max	M (P_25_,P_75_)	Min	Max	M (P_25_,P_75_)	Min	Max	M (P_25_,P_75_)
Before treatment	280.14	2711.19	1469.16 (367.50, 2610.58)	717.98	4206.48	1904.30 (964.51, 3681.00)	2358.86	3425.01	3013.82(2358.86, -)
6 months after treatment	123.95	1249.03	545.90 (133.39, 1169.30)	110.45	1001.60	735.58 (234.98, 966.85)	124.55	854.76	784.39(124.55, -)
**Volume difference rate of the bilateral limbs before and after lymphedema treatment**									
Time point	Min	Max	M (P_25_,P_75_)	Min	Max	M (P_25_,P_75_)	Min	Max	M (P_25_,P_75_)
Before treatment	0.04	0.44	0.22 (0.05, 0.42)	0.17	0.58	0.30 (0.20, 0.51)	0.39	0.68	0.43 (0.39, -)
6 months after treatment	0.02	0.20	0.08 (0.02, 0.19)	0.03	0.14	0.12 (0.05, 0.14)	0.02	0.17	0.11 (0.02, -)
**Lymph-ICF-LL score before and after lymphedema treatment (points)**									
Time point	Min	Max	M (P_25_,P_75_)	Min	Max	M (P_25_,P_75_)	Min	Max	M (P_25_,P_75_)
Before treatment	150	161	155 (150, 160)	142	200	168 (146, 195)	153	189	158 (153, -)
6 months after treatment	42	73	56 (45, 69)	39	76	66 (44, 76)	43	62	59 (43, -)

CDT, complex decongestive therapy; LVA, lymphovenous anastomosis; Lymph-ICF-LL, Lymphedema Functioning, Disability and Health Questionnaire for Lower Limb Lymphedema.

All patients with lymphedema who received CDT treatment and surgical intervention showed reduced limb volume, particularly after liposuction. However, one patient experienced a postoperative complication of localized skin necrosis (approximately 4 cm^2^) after the liposuction procedure, while others did not develop complications such as lymphatic leakage, wound dehiscence, or infection. During the follow-up period, all patients showed an improvement in their quality of life. Lastly, all patients wore compression garments with a pressure of 30–40 mmHg to maintain the therapeutic effect.

## Discussion

In this study, six patients with Stage I or IIa lymphedema complicated by iliac vein compression exhibited varying degrees of edema relief following iliac vein stenting. Although these patients derived some short-term benefits from the procedure, all experienced recurrence and further progression of limb swelling. The remaining five lymphedema patients showed no improvement in limb edema after iliac vein stenting. Notably, all patients demonstrated reductions in limb volume and improved quality of life after receiving CDT treatment or undergoing lymphedema-related surgical interventions, particularly following liposuction procedures. Therefore, for patients presenting with lower limb edema and radiologically confirmed iliac vein compression, clinicians should thoroughly inquire about potential histories of radical surgery for gynecological malignancies or other surgical procedures. Subsequently, differential diagnosis of lymphedema should be considered for patients with relevant medical histories, and stent implantation should be approached with greater caution.

Prior cadaveric and radiographic studies have reported that the prevalence of iliac vein compression is remarkably high in the general population, with a particular predilection for women aged 30–50 years, whose clinical manifestations include limb swelling [4,5,8]. Previous studies have reported that approximately 50% of patients develop lower extremity lymphedema ≥5 years after receiving gynecologic cancer treatment [14]. The age of postoperative lymphedema onset in gynecological malignant tumors is also approximately 50 years of age, which highlights the need to distinguish it from MTS characterized by limb swelling.

In cases where iliac vein compression is detected through imaging examinations, the decision to perform stent implantation depends on the degree of stenosis, clinical symptoms, and Clinical, Etiology, Anatomy, and Pathophysiology (CEAP) classification [15]. According to the guidelines proposed by the American Venous Forum, Society for Vascular Surgery, European Society for Vascular Surgery, and Society of Interventional Radiology, stent implantation is recommended for patients who have clinically relevant venous outflow obstruction classified as CEAP 3–6, along with venous claudication, pelvic pain, and morphological indication of >50% area reduction [16–18]. Stenting may be considered for patients with edema due to venous disease (i.e., CEAP 3), provided careful clinical judgment is exercised because of the potential for varied coexisting nonvenous causes of edema. However, the recommendations in the previously mentioned guidelines are all based on venous-related diseases. The detection of iliac vein stenosis on imaging examinations in patients with lymphedema may indicate venous-lymphatic mixed edema rather than purely venous-related edema. Therefore, stent implantation should be considered with even greater caution in such patients. However, patients with Stage I and II lymphedema and concurrent significant iliac vein compression on venography examination may still achieve some benefits through iliac vein stent implantation. Thus, the rationality of this procedure cannot be completely negated. Relief of venous obstruction can also improve lymphatic stagnation to some extent. Moreover, subsequent LVA requires a relatively normal venous return status and cannot be performed under the conditions of an obstructed venous outflow tract. Furthermore, veins with good valve function should be selected to avoid venous reflux, which can cause thrombosis at the anastomotic site between the lymphatic ducts and veins [19,20,21].

In the present study, patients with lymphedema were initially treated using conservative therapy, specifically systematic CDT. Conservative therapies, such as the application of short-stretch bandages, compression garments, and manual lymphatic drainage, constitute the first-line treatment for lymphedema of the extremities [9]. Most patients, particularly those with early-stage lymphedema, can achieve satisfactory results with CDT. LVA serves as an effective treatment option in patients with Stage I or IIa lymphedema who have failed conservative treatment, experienced recurrent lymphangitis, or are highly dependent on compressive therapy measures. LVA is a microsurgical technique that creates a bypass between submillimeter-sized lymphatic channels and veins to improve lymphatic drainage, reduce edema volume, and alleviate related symptoms [22,23]. LVA is a safe and effective treatment method, which is minimally invasive and does not cause severe complications [24,25]. In the case of patients with Stage IIb or III lymphedema characterized by substantial fat deposition, liposuction is employed to rapidly reduce limb volume, followed by inguinal LVA in the second stage to augment lymphatic flow. The satisfactory treatment outcomes observed in the patients with lymphedema in this study can be attributed to the appropriate surgical options implemented based on their distinct pathophysiological changes [24,25].

This study has several important limitations that should be acknowledged. First, the majority of included patients received iliac venous stent implantation at external institutions, resulting in insufficient standardized data for precise pre- versus post-stenting limb morphological evaluation. Therefore, this study compared the persistent limb swelling following stent implantation with the clinical outcomes after specialized lymphedema treatment. Second, the inherent clinical rarity of this condition led to a constrained sample size that may limit statistical power. Finally, the present study exclusively enrolled referred cases with suboptimal clinical outcomes following stent implantation, which may introduce potential selection bias that could limit the generalizability of the findings.

### Ethics approval

This study was conducted by the principles outlined in the Declaration of Helsinki. Approval was granted by the Ethics Committee of Suzhou Municipal Hospital (IRB Number: J-2024–042-K01, dated November 25, 2024). Informed consent was obtained from all individual participants included in the study. Patients signed informed consent forms regarding the publication of their data and photographs.

## Conclusion

In patients with secondary lymphedema complicated by iliac vein compression, the indications for iliac vein stenting should be more strictly controlled. LVA or liposuction, based on the different pathophysiological stages of secondary lymphedema, can achieve favorable therapeutic outcomes.

## Supporting information

S1 Data. (XLSX)
